# Enhancing the Substrate Specificity of *Clostridium* Succinyl-CoA Reductase for Synthetic Biology and Biocatalysis

**DOI:** 10.1021/acs.biochem.3c00102

**Published:** 2023-05-19

**Authors:** Pascal Pfister, Christoph Diehl, Eric Hammarlund, Martina Carrillo, Tobias J. Erb

**Affiliations:** † Department of Biochemistry & Synthetic Metabolism, Max Planck Institute for Terrestrial Microbiology, Karl-von-Frisch Str. 10, 35043 Marburg, Germany; ‡ SYNMIKRO Center for Synthetic Microbiology, Karl-von-Frisch Str., 14, 35032 Marburg, Germany

## Abstract

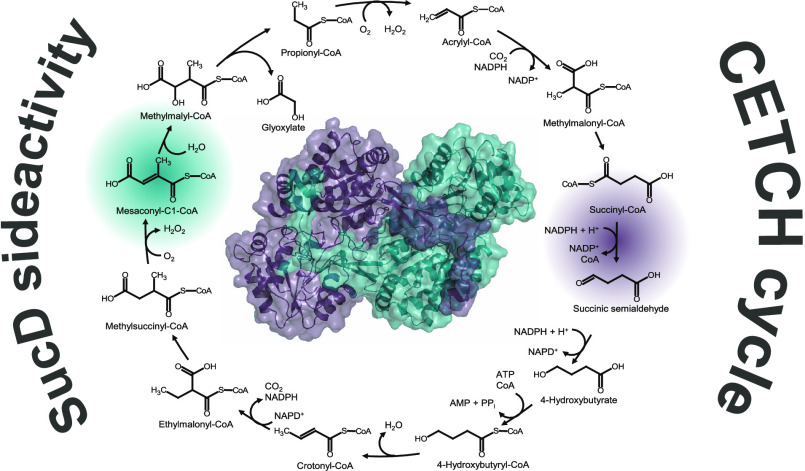

Succinyl-CoA reductase (SucD) is an acylating aldehyde reductase that catalyzes the NADPH-dependent reduction of succinyl-CoA to succinic semialdehyde. The reaction sequence from succinate to crotonyl-CoA is of particular interest for several new-to-nature CO_2_-fixation pathways, such as the crotonyl-CoA/ethylmalonyl-CoA/hydroxybutyryl-CoA (CETCH) cycle, in which SucD plays a key role. However, pathways like the CETCH cycle feature several CoA-ester intermediates, which could be potentially side substrates for this enzyme. Here, we show that the side reaction for most CETCH cycle metabolites is relatively small (<2%) with the exception of mesaconyl-C1-CoA (16%), which represents a competing substrate in this pathway. We addressed this promiscuity by solving the crystal structure of a SucD of *Clostridium kluyveri* in complex with NADP^+^ and mesaconyl-C1-CoA. We further identified two residues (Lys70 and Ser243) that coordinate mesaconyl-C1-CoA at the active site. We targeted those residues with site-directed mutagenesis to improve succinyl-CoA over mesaconyl-C1-CoA reduction. The best resulting SucD variant, K70R, showed a strongly reduced side activity for mesaconyl-C1-CoA, but the substitution also reduced the specific activity for succinyl-CoA by a factor of 10. Transferring the same mutations into a SucD homologue from *Clostridium difficile* similarly decreases the side reaction of this enzyme for mesaconyl-C1-CoA from 12 to 2%, notably without changing the catalytic efficiency for succinyl-CoA. Overall, our structure-based engineering efforts provided a highly specific enzyme of interest for several applications in biocatalysis and synthetic biology.

## Introduction

**Figure 1:**
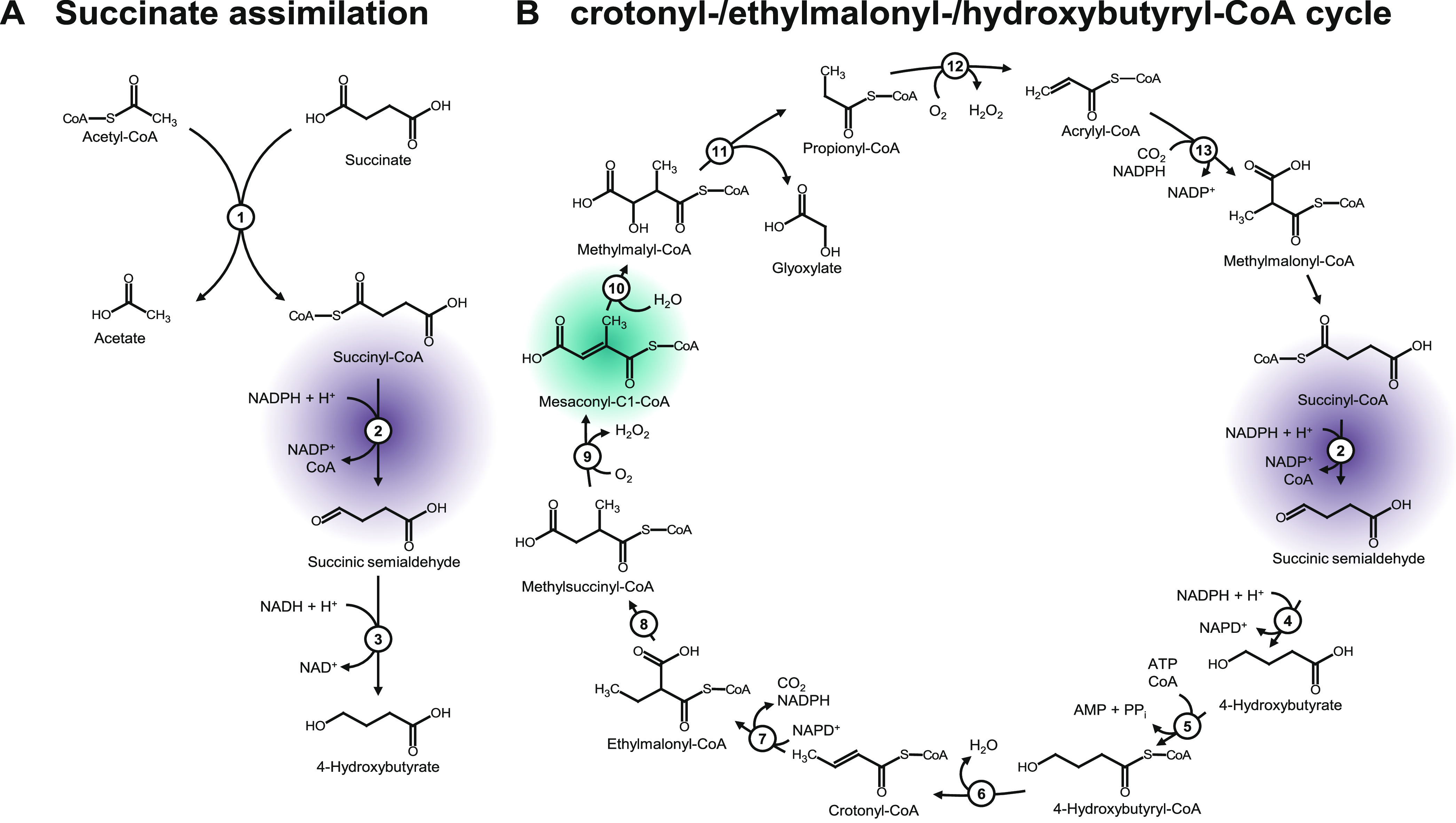
Succinate assimilation pathway of *C. kluyveri*.^1,11^ The enzymes, undefined CoA transferase (1, Cat1, and Cat2), succinyl-CoA reductase (2, SucD), and 4-hydroxybutyrate dehydrogenase (3, 4Hbd), mediate the conversion of succinate to 4-hydroxybutyrate. The coassimilation steps of ethanol to balance reduction equivalents are not shown (A). The reactions of the crotonyl-CoA/ethylmalonyl-CoA/hydroxybutyryl-CoA (CETCH) cycle (B);^8^ succinic semialdehyde reductase,^4^ 4-hydroxybutytyl-CoA synthetase,^5^ 4-hydroxybutytyl-CoA dehydratase,^6^ enoyl-CoA carboxylase/reductase (7 + 13) ethylmalonyl-CoA mutase,^8^ methylsuccinyl-CoA oxidase,^9^ mesaconyl-C1-CoA hydratase,^10^ β-methylmalyl-CoA lyase,^11^ and propionyl-CoA oxidase.^12^ The reaction of SucD is highlighted in purple. The alternative substrate mesaconyl-C1-CoA is highlighted in teal.

Succinyl-CoA reductase (SucD) is an acylating aldehyde reductase that catalyzes the NADPH-dependent reduction of succinyl-CoA to succinic semialdehyde (SSA). In *Clostridium kluyverii* and other *Clostridia* species, succinyl-CoA reductase operates in fatty acid fermentation, allowing for the co-assimilation of ethanol and succinate.^1−6^ In these fermentations, succinate is first activated to succinyl-CoA, which is then reduced to SSA by SucD before being further converted into 4-hydroxybutyrate (see Figure 1).

The reaction mechanism of SucD supposedly follows the canonical reaction mechanism of acylating aldehyde dehydrogenases.^7^ In these enzymes, the respective acyl-CoA enters the active site, where the acyl moiety is transferred onto an active site cysteine, leading to a covalent acyl-cysteine intermediate. The freed CoA moiety is protonated by a nearby histidine before exiting the active site, while the acyl moiety is reduced to the aldehyde by NADPH.

Beyond its role in the fermentation of short-chain fatty acids, SucD has found increasing attention for the realization of artificial CO_2_ fixation pathways. The most prominent example is the CETCH (crotonyl-CoA/ethylmalonyl-CoA/hydroxybutyryl-CoA) cycle, which has been established recently.^8^ The CETCH cycle is a complex pathway for the assimilation of CO_2_.^8^ It involves 17 different enzymes that were re-wired to form a new-to-nature pathway for the capture and conversion of CO_2_. This pathway was reconstituted *in vitro*,^8^ optimized through rational and machine-learning approaches,^9^ and connected with downstream biosynthetic modules to produce different value-added compounds like polyketides and terpenes directly from CO_2_.^10^

Key to the successful realization of new-to-nature pathways is the selection and/or design of suitable catalysts with exquisite substrate specificity and little promiscuity. Especially for the construction of complex new-to-nature networks that share structurally similar metabolites, such as the CETCHcycle, high substrate specificity is essential to avoid draining of metabolites and accumulation of dead-end products, which ultimately lead to stalling of the metabolic network.^8^ To refeed side products into the reaction sequence, additional enzymes can be added (metabolic proofreading).^8^ However, the side reaction of SucD produces unfavorable semialdehyde products, which are metabolically hard to proofread. As the *in vitro* constitution of the CETCH cycle as well as a potential *in vivo* has a constant recovery of NADPH, both reductions are virtually irreversible.

In this study, we assessed the substrate specificity of SucD from *C. kluyverii* (CkSucD) for its use in synthetic biology. We show that CkSucD is catalytically selective for many CoA-esters with a relative catalytic efficiency (*k*_cat_/*K*_M_) below 2%, but has a significant side activity with mesaconyl-C1-CoA (16%), which is an important intermediate of both, the CETCH and the THETA cycle. We solved the crystal structure of SucD with mesaconyl-C1-CoA to identify amino acids conferring substrate binding at the active site. We further targeted these residues to improve the substrate specificity of CkSucD and transferred the best mutation into the scaffold of SucD from *Clostridium difficile* (CdSucD) to obtain a highly specific SucD variant.

## Materials and Methods

### Plasmid Generation

The plasmid containing His-tagged SucD of *C. kluyverii* (pTE380)^8^ and *C. difficile* (pTE1816)^12^ was used as described in previous studies. Plasmids with point mutations were generated by site-directed mutagenesis with a single primer depicted in Table 1.^13^

**Table 1:** Primers Used in This Study

primer	sequence
PCC_E_seq. 1	CTTATGCGACTCCTGCATTAGG
pDuet_primus_rev	CGATTATGCGGCCGTGTACAATACG
*SucD_Ck_K66R*	GTTTATGAAGATAAAGTAGCTAGATGTCATTTGAAATCAGGAGC
*SucD_Ck_K70R*	GTTTATGAAGATAGAGTAGCTAAATGTCATTTGAAATCAGGAGC
*SucD_Ck_K66R_K70R*	GTTTATGAAGATAGAGTAGCTAGATGTCATTTGAAATCAGGAGC
*SucD_Ck_S243N*	CAATGGAATTATATGTAATTCAGAGCAATCAGTTATAGCTCCTGC
*SucD_Ck_T112F*	GCTACTACGCCTATATTTAATCCAGTGGTAACTC
*SucD_Cd_K79R*	GAAAAACAAGTCTAGGGCGAAGGTGATC

### Synthesis of CoA Esters

CoA ester synthesis was performed according to published protocols.^14,15^

### Gene Expression and Protein Purification

Chemical competent *Escherichia coli* BL21 DE3 cells were transformed with expression plasmids and selected for on LB agar plates using the respective antibiotics. For protein production, cells containing the plasmids were cultivated in 1 L of salt-buffered TB medium at 100 r.p.m and a temperature of 37 °C until the culture reached an OD_600_ of 0.8. Afterward, flasks were transferred to a shaker at 18 °C, and cells were induced with 0.25 mM IPTG and grown overnight. Cells were harvested for 10 min at 4 °C and 5000*g*. The pellet was re-suspended in a 3-fold volume (3 mL per 1 g of cells) in lysis buffer (50 mM HEPES/KOH pH 7.8, 500 mM NaCl, 10% glycerol) and 10 μg/mL DNAse, and 5 mM MgCl_2_ was added. Cells were lysed by ultrasonication. The membrane fractions were removed by centrifugation at 18 000*g* and 4 °C for 1 h. The lysate was filtered through a 0.45 μm syringe filter before loading on a 1 mL HisTrap FF (pre-equilibrated with lysis buffer) column attached to an Äkta Start (both from GE Healthcare, Freiburg, Germany). Unspecific bound proteins were washed off using lysis buffer with 75 mM imidazole. The bound protein was eluted from the column using lysis buffer with 500 mM imidazole and collected in 1 mL fractions.

The eluted fractions were desalted using 2 × 5 mL HiTrap desalting columns (GE Healthcare, Freiburg, Germany) equilibrated with desalting buffer (50 mM HEPES/KOH, pH 7.8, 200 mM NaCl, 10% glycerol). The fractions containing the protein of interest were pooled and concentrated using Amicon Ultra-4 centrifugal filters (Merck Millipore, Darmstadt, Germany). For storage, glycerol was added to a final concentration of 20% and aliquots were frozen in liquid nitrogen before storing them at −80 °C.

For crystallization, the desalted proteins were further purified using size exclusion chromatography. Therefore a HiLoad 16/600 Superdex 200 pg attached to an Äkta Pure (both from GE Healthcare, Freiburg, Germany) was equilibrated with desalting buffer containing 20 mM TRIS-HCl, pH 7.8, 50 mM NaCl. The fractions containing the protein were pooled and concentrated in Amicon Ultra-15 centrifugal filters (Merck Millipore, Darmstadt, Germany), which were washed in advance to remove glycerol from the membranes.

### Enzymatic Assays

Reduction rates of CoA thioesters were determined by spectrophotometric monitoring of NADPH oxidation. A reaction mixture of 300 μL (200 mM HEPES, pH 7.5, 400 μM NADPH, 400 nM CkSucD) was incubated at 30 °C for 1 min. Varying amounts of CoA thioesters were added to the mixture to start the reaction. NADPH oxidation was monitored at a wavelength of 365 nm using an extinction coefficient of 3300 M^–1^ cm^–1^^16^ in order to allow higher concentrations of NADPH. For specific activity measurements, 1 mM succinyl- or mesaconyl-C1-CoA were used.

### Crystallization and Structure Determination

The purified protein solution was spotted in different concentrations (5 and 10 mg mL^–1^) on sitting-drop vapor-diffusion crystallization plates. 0.2 μL of each protein solution was mixed with 0.2 μL of crystallization condition (for PDB 8CEI—25% PEE propoxylate, 100 mM MES, pH 6.5, 50 mM MgCl_2_; for PDB 8CEK—45% PEE propoxylate, 100 mM MES, pH 6.5, 400 mM KCl, 5 mM NADPH; for PDB 8CEJ—100 mM magnesium acetate, 100 mM MOPS, pH 7.5, 5 mM mesaconyl-C1-CoA, 12% PEG 8000). The drops equilibrated against 30 μL of protein free crystallization condition at 288 K. Crystals formed within 24–48 h. A cryoprotectant was added to the crystals to exceed a final concentration of 40% v/v (PEG 200 for PDB 8CEJ and PEE propoxylate for PDB 8CEI and 8CEK respectively). For crystals of PDB 8CEJ, mesaconyl-C1-CoA was added to 5 mM, additionally. All crystals were looped and frozen in liquid nitrogen. X-ray diffraction data were collected at the beamline P13 of the Deutsches Elektronen–Synchrotron (DESY). The data sets were processed with the XDS software package.^17^ The structures were solved by molecular replacement using a poly alanine search model of a probable aldehyde dehydrogenase from *Listeria monocytogenes* (PDB ID 3K9D). Molecular replacement was carried out using Phaser of the Phenix software package^18^ and refined with Phenix.Refine. Additional modeling, manual refining, and ligand fitting were done in COOT.^19^ Final positional and B-factor refinements as well as water-picking for the structure were performed using Phenix.Refine. The structure models were deposited at the PDB in Europe under PDB IDs 8CEJ, 8CEI, and 8CEK. Data collection and refinement statistics are provided in Table 2.

**Table 2:** Data Collection and Refinement Statistics of CkSucD Structures^a^

PDB ID	8CEI	8CEK	8CEJ
ligands		NADPH	mesaconyl-C1-CoA, mesaconate
wavelength	0.97620	0.97620	0.97630
resolution range (Å)	39.5–2.2 (2.3–2.2)	29.7–2.1 (2.2–2.1)	24.6–2.1 (2.2–2.1)
space group	*P*12_1_1	*I*222	*I*222
unit cell dimensions			
*a*, *b*, *c* (Å)	86.2, 89.3, 137.3	140.0, 190.8, 190.9	141.3, 189.7, 189.5
α, β, γ (deg)	90.0, 104.6, 90.0	90.0, 90.0, 90.0	90.0, 90.0, 90.0
total reflections	449 228 (62 718)	2 045 343(169 254)	1 951 988 (296 160)
unique reflections	10 2176 (14 359)	159 239 (21 156)	147 479 (21 387)
multiplicity	4.4 (4.4)	12.8 (8.0)	10.9 (4.1)
completeness (%)	99.19 (98.07)	99.85 (99.89)	99.83 (99.90)
mean *I*/σ(*I*)	7.3 (2.7)	8.5 (2.6)	10.9 (4.1)
R-merge	0.102 (0.411)	0.207 (0.275)	0.152 (0.740)
R-pim	0.053 (0.558)	0.059 (0.966)	0.044 (0.207)
CC1/2	0.995 (0.945)	0.998 (0.718)	0.997 (0.932)
reflections used in refinement	101 689 (10 008)	138 025 (13 637)	147 363 (14 634)
R-work	0.2424 (0.3033)	0.1980 (0.2902)	0.2949 (0.3344)
R-free	0.2670 (0.3073)	0.2221 (0.3261)	0.3095 (0.3281)
number of non-hydrogen atoms	14 600	14 888	15 096
macromolecules	13 576	13 630	13 666
ligands	0	192	42
solvent	1024	1066	1388
protein residues	1788	1795	1794
RMS(bonds)	0.002	0.004	0.004
RMS(angles)	0.40	0.58	0.43
Ramachandran			
favored (%)	98.31	98.15	97.08
allowed (%)	1.52	1.62	2.59
outliers (%)	0.17	0.22	0.34
Rotamer outliers (%)	0.83	0.62	0.48
Clashscore	2.71	2.41	2.00
Average B-factor	33.59	47.07	29.29
macromolecules	33.40	46.70	29.05
ligands		67.05	27.29
solvent	36.12	48.11	31.76
Twin fraction (law)			0.4 (-h, -l, -k)

a Statistics for the highest-resolution shell are shown in parentheses.

### Structural Modeling of CdSucD and CkSucD Mutants

A structure model of CdSucD was generated using the software package SWISS-MODEL (www.swissmodel.expasy.org)^20−22^ by providing PDB 8CEJ and the respective sequence files.

## Results

### SucD from *C. kluyveri* Is Promiscuous with Mesaconyl-C1-CoA

To investigate the substrate specificity of SucD, we determined the activity of CkSucD with its native substrate, succinyl-CoA, and different alternative CoA esters (Table 3). CkSucD displayed a *k*_cat_/*K*_M_ of 3.5 × 10^5^ M^–1^ s^–1^ for succinyl-CoA and 2% or less catalytic efficiency for most other CoA esters. One notable exception was mesaconyl-C1-CoA, which showed a catalytic efficiency of 5.6 × 10^4^ M^–1^ s^–1^, corresponding to 16% of its native reaction with succinyl-CoA. Overall, this data indicated that the use of CkSucD could be problematic with pathways featuring mesaconyl-C1-CoA as a metabolite, such as the CETCH or THETA cycle.

**Table 3:**
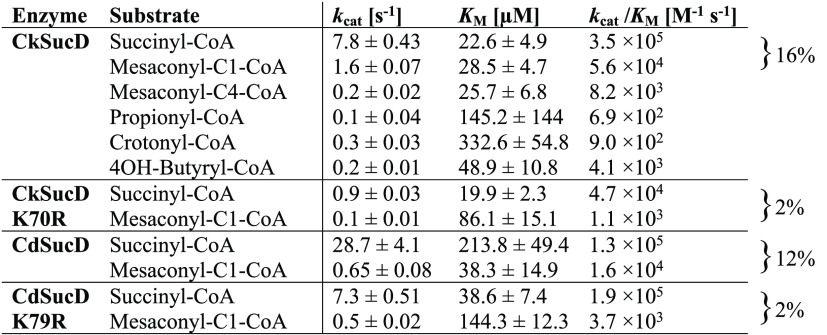
Kinetic Parameters of SucD Variants^a^

a “±” indicates SE. Fits of Michaelis–Menten kinetics are shown in Figures 4 and S3.

### Crystal Structure of CkSucD Identifies Molecular Basis for Mesaconyl-C1-CoA Binding

**Figure 2:**
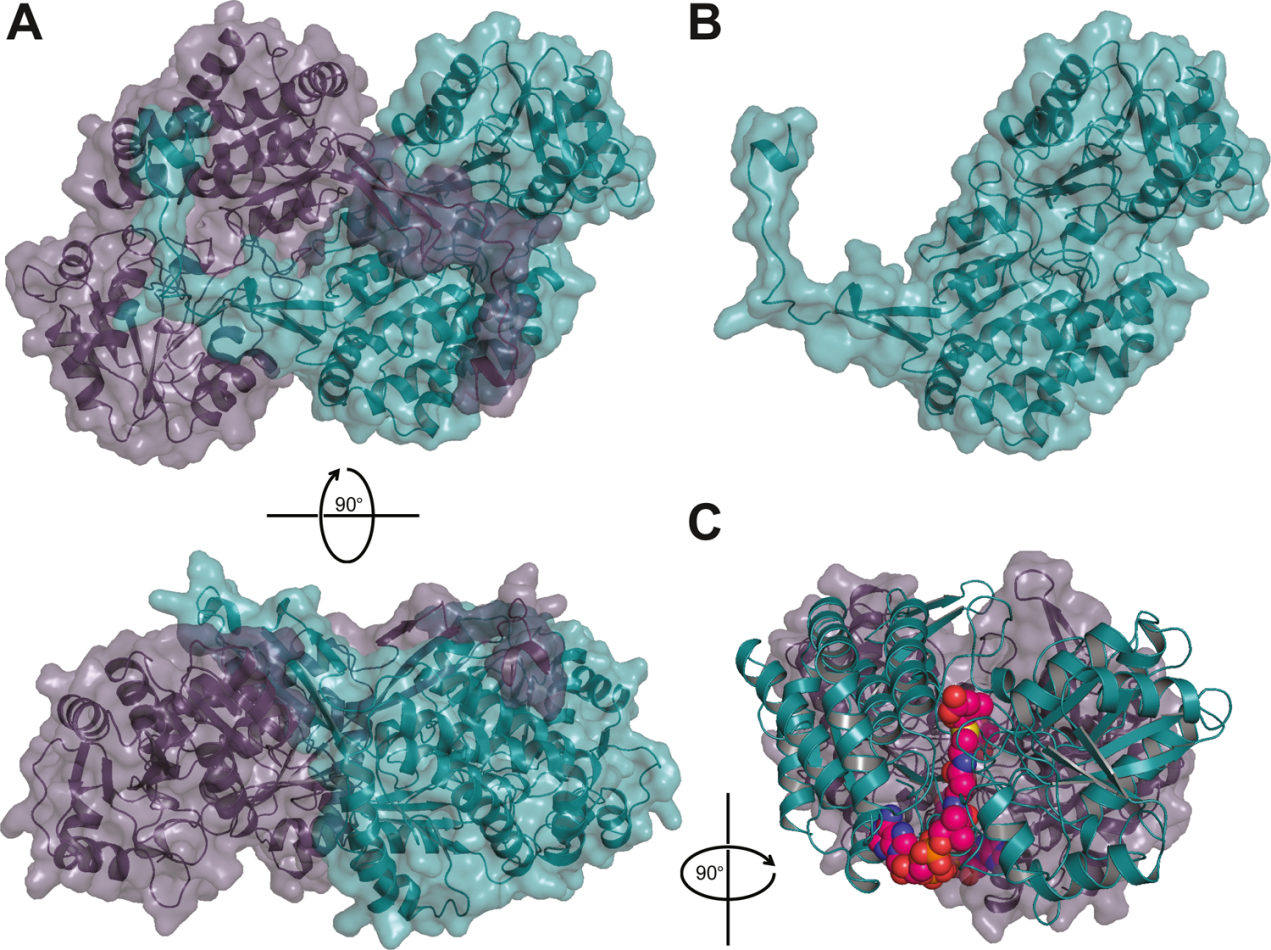
Structure of CkSucD with bound ligands; CkSucD (pdb 8CEI) forms a homodimer (A) with an overlapping C-terminal loop (B). CoA ester substrates cross the whole subunit (pdb 8CEJ) to reach toward the active site. Mesaconyl-C1-CoA is shown in pink (C).

To understand the basis of substrate specificity in CkSucD, we solved the crystal structure of the enzyme in complex with mesaconyl-C1-CoA. CkSucD forms a homodimeric complex (Figure 2A). Each monomer (Figure 2B) has an extended C-terminal loop that reaches into the second subunit within the complex. CoA ester ligands are coordinated within a tubular cavity that reaches to the surface bound active site on the other side of the monomer (Figure 2C).

**Figure 3:**
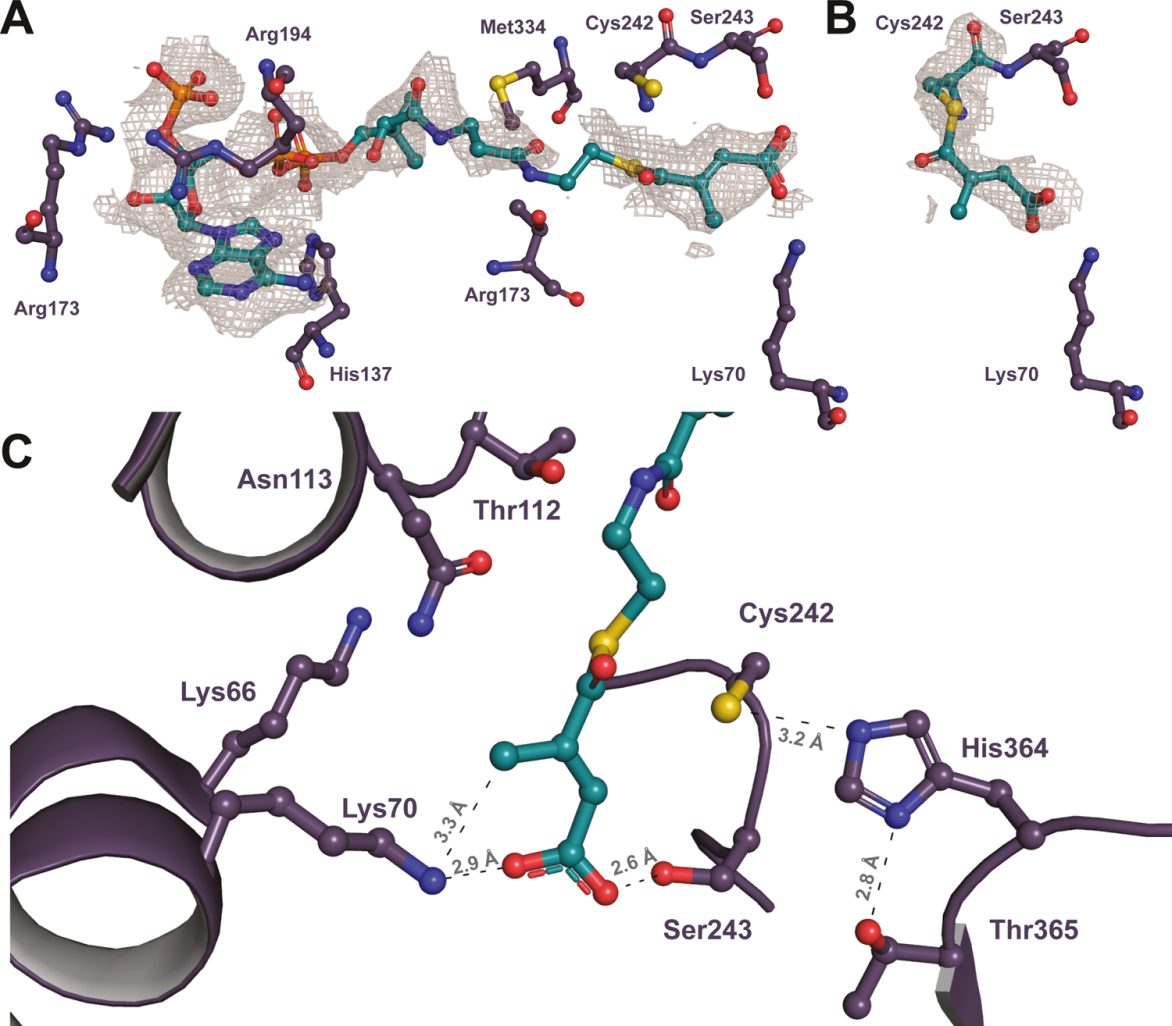
Active site of CkSucD. The acyl moiety of mesaconyl-C1-CoA is coordinated by Lys70 and Ser243 (A). Cys242 forms a mesaconyl-cysteine intermediate (B). Thr365 and His364 donate a proton to form the product (C). Peptide residues are presented in violet, and mesaconylated intermediates are presented in teal. The mesh represents a simulated annealing omit map (*F*_o_ – *F*_c_) at 2.0 σ.

The secondary structure of CkSucD shares high similarity to and PduP from *Rhodopseudomonas palustris* (pdb 5JFN, Figure S2A, RMSD 1.031 over 302 residues) and is—at 32% identities—the closest protein structure with a trapped intermediate.^7,23^ The active site of propionaldehyde dehydrogenases is identical in respect to the mechanistically relevant residues but differs in the active site surroundings (Figure S2B). Compared to PduP, residues that restrict the active site pocket and coordinate the acyl moiety of propionyl-CoA (Leu158, Leu483, and Val331) are absent in CkSucD (aligned residues in CkSucD are Lys70, Thr395, and Ser243), which creates a pocket that is differently shaped and more spacious^4,7^ (Figure S2A). Mesaconyl-C1-CoA is coordinated through Ser243, which is located next to the catalytically active His242 and forms a hydrogen bond to the carboxyl group of mesaconyl-C1-CoA. The terminal carboxyl group of mesaconyl-C1-CoA is further coordinated through a hydrogen bond to Lys70 (Figure 3C).

The reaction mechanism of CkSucD is likely analogous to that of propionaldehyde dehydrogenases (PduP),^7^ as it shares the aforementioned active site cysteine, histidine, and threonine in direct proximity to the CoA ester and mesaconyl-cysteine intermediate (Figures 3B and S2B). An active site cysteine (Cys242) plays a key role by forming a covalent bond with the acyl moiety of the CoA ester and releasing the CoA moiety (Figure 3B). Active site residues His364 and Thr365 assist in proton donation to the released CoA (Figure 3C). In our structure, we observed mesaconyl-C1-CoA coordinated at the active site (Figure 3A) with a occupancy of 75% (pdb 8CEJ, C). However, in the rest of the subunits, the electron density allowed for the assignment of a mesaconyl-cysteine (pdb 8CEJ, A,B,D), indicating that we also trapped the covalent intermediate of the reaction mechanism in part of our crystals.

### Active Site Mutagenesis to Increase the Selectivity of CkSucD for Succinyl-CoA

Based on our structures, we identified several residues that we targeted to increase the selectivity (relative catalytic efficiency, lower is more selective) of CkSucD to avoid mesaconyl-C1-CoA reduction. To discriminate sterically and electronically against the methyl group of mesaconyl-C1-CoA, we replaced Lys70 by a bulkier, positively charged arginine. For the same reason, we also exchanged Lys66, which is located on the same α-helix as Lys70, by an arginine. We also mutated Ser243 to an asparagine to allow for hydrogen bonding to the mesaconyl-C1-CoA carboxyl group, while increasing steric constraints against the methyl group. Finally, we introduced a phenylalanine at the position of peripheral Thr112, which coordinates the amide group of the cysteamine in the CoA moiety and controls access to the active site, with the idea of restricticting mesaconyl-CoA accommodation.

All single mutants were soluble but showed only residual or nondetectable activity with succinyl-CoA (Table 4). Only variants K70R and K66R showed relevant turnover rates, albeit at one-tenth and one-twentieth of wild-type activity, respectively. Double variants K66R_K70R and K70R_ S243N did result in insoluble or nonactive protein, which left us with the K70R mutant, as this variant also had shown some improved specificity (8% relative activity of mesaconyl-C1-CoA to succinyl-CoA reduction) compared to wild-type (16%) and the K66R variant (34%) in our screen.

**Table 4:** Specific Activities (s) of Different CkSucD Mutants^a^

		specific activity [s^–1^]		
mutation	solubility	succinyl-CoA	mesaconyl-C1-CoA	side activity [%]
wild-type	+	7.8 ± 0.43	1.6 ± 0.07	20.5
K66R	+	0.48 ± 0.02	0.164 ± 0.01	34.2
K70R	+	0.84 ± 0.014	0.072 ± 0.01	8.6
S243N	+	n.d.	n.d.	
T112F	+	0.10 ± 0.001	0.03 ± 0.003	30
K66R_K70R	−	−	−	−
K70R_S243N	+	n.d.	n.d.	

a “±” indicates SE and “n.d.” not detectable.

### CkSucD K70R Shows Increased Selectivity, Albeit at 10-fold Reduced Catalytic Efficiency for Succinyl-CoA

**Figure 4:**
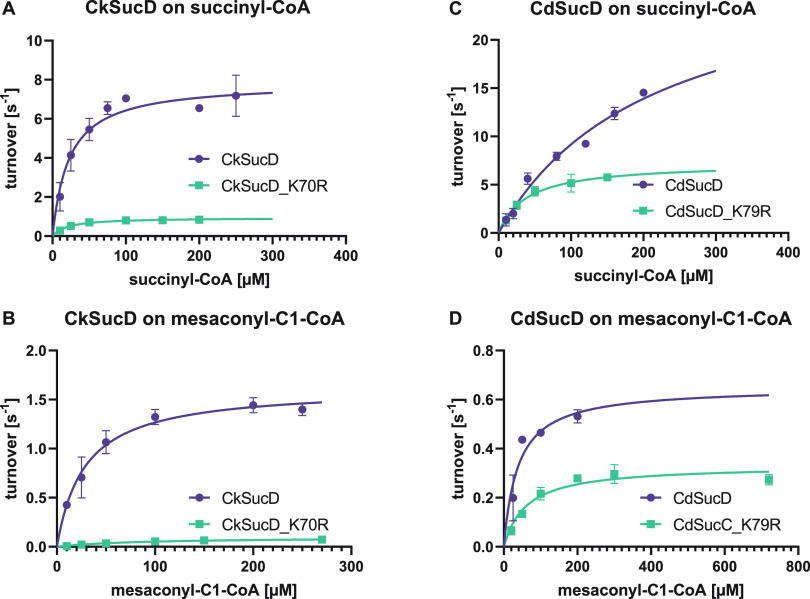
Michaelis–Menten kinetics of SucD variants. Activities of CkSucD and the K70R variant on succinyl-CoA and mesaconyl-C1-CoA (A + B). Activities of CdSucD and the K79R variant on both substrates (C + D).

The kinetic parameters of CkSucD K70R for succinyl-CoA and mesaconyl-C1-CoA were determined to compare with those of the wild-type (Table 3 and Figure 4). The catalytic activity of active site variant CkSucD_K70R for succinyl-CoA was reduced by one order of magnitude (from ∼4 × 10^5^ to ∼5 × 10^4^ M^–1^ s^–1^). However, the catalytic efficiency for mesaconyl-C1-CoA reduction had dropped more than 50-fold (from ∼6 × 10^4^ to ∼1 × 10^3^ M^–1^ s^–1^). This was caused by an 3-fold increased apparent *K*_M_ values of for mesaconyl-C1-CoA (from ∼30 to ∼90 μM), while the drop in specific activity contributed by roughly a factor of two. Together, these factors decreased the relative catalytic efficiency of K70R with mesaconyl-C1-CoA from 16 to 2%; yet, this specificity increase came at a 10-fold decreased catalytic efficiency for the original substrate.

### *C. difficile* SucD K79R Shows Increased Selectivity at a High Catalytic Efficiency

We also tested the effects of the K70R mutation could be transferred to other homologues. To that end, we introduced the equivalent substitution (K79R) into SucD from *C. difficile* (CdSucD). Notably, this mutation did not negatively affect the catalytic efficiency of the reaction with succinyl-CoA (catalytic efficiency was actually slightly increased), while the catalytic efficiency with mesaconyl-C1-CoA dropped more than 4-fold. This was mainly caused by a 3-fold increased apparent *K*_M_ for mesaconyl-C1-CoA. Overall, the K79R mutation reduced the relative catalytic efficiency with mesaconyl-C1-CoA to about 2%, while the catalytic efficiency for the native substrate remained virtually unchanged.

The apparent *K*_M_ value of CkSucD for succinyl-CoA was barely affected by the K70R mutation, while the corresponding value for CdSucD decreased notably from 210 to 40 μM as a result of the K79R substitution. Homology modeling of CdSucD on CkSucD (Figure S4) does not show significant differences with respect to the topology between both active sites. However, the overall amino acid sequence identity between both isoenzymes is only 58%, which may cause differences in the dynamic organization of the active site during catalysis. These differences cannot be predicted by homology modeling, which could explain the decreased *K*_M_ for succinyl-CoA in CdSucD but not in CkSucD upon introduction of the arginine.

## Discussion

In this study, we investigated the substrate specificity of SucD, an essential enzyme in ethanol-succinate fermentation and a key enzyme in several new-to-nature CO_2_ fixation pathways that were developed recently.^8^ We show that the enzyme is not only an efficient succinyl-CoA reductase with a *k*_cat_ of 7.8 ± 0.43 s^–1^ but also possesses a significant side activity with mesaconyl-CoA at 16% specific activity.

To understand the molecular basis of this promiscuity, we solved the crystal structure without ligands (pdb 8CEI), as well as in the NADPH- (pdb 8CEK) and mesaconyl-C1 CoA bound state (pdb 8CEJ, which also includes a catalytically trapped mesaconate-cysteine intermediate). Our structures at a resolution between 2.1 and 2.2 Å (re-)confirm catalytically active residues and residues necessary for the coordination and binding of mesaconyl-C1-CoA. Ser243 and Lys70, which we subsequently targeted for site-directed mutagenesis, coordinate the distal carboxyl group of mesaconyl-C1-CoA.

To engineer the substrate specificity of CkSucD, we created different active site mutants, of which K70R decreased the relative catalytic efficiency with mesaconyl-C1-CoA from 16 to 2% yet, this mutation also decreased the catalytic efficiency for succinyl-CoA by 10-fold. When transferring this mutation into the closely related homolog CdSucD, relative catalytic efficiency dropped again to 2%, notably, however, without affecting the catalytic activity with succinyl-CoA, yielding a highly specific, yet highly active, enzyme. Note that this 2% side reactivity represents an upper limit, as the selectivity might even increase, especially in situations in which the enzyme faces low concentrations of the respective CoA esters, as the apparent *K*_M_ for mesaconyl-C1-CoA (∼145 μM) is considerably higher than for succinyl-CoA (∼40 μM).

Overall, the CdSucD_K79R variant created in this study is a highly specific succinyl-CoA reductase for future use in the construction and operation of new-to-nature pathways, such as the CETCH cycle, as well as other metabolic networks featuring mesaconyl-C1-CoA as a metabolite.

## Supporting Information Available

Overlay of the active site of CkSucD with models of CkSucD_K70R, PduP, and CdSucD; Michaelis–Menten kinetics of CkSucD for different acyl-CoAs (PDF)

## Accession Codes

PDB-IDs for CkSucD are 8CEI (without ligands), 8CEK (with NADPH), and 8CEJ (with mesaconyl-C1-CoA). Uniprot IDs are P38947 for CkSucD and A0A031WJ42 for CdSucD.
