# Supplementary material for: Enhancing the Substrate Specificity of Clostridium Succinyl-CoA Reductase for Synthetic Biology and Biocatalysis
Source: Biochemistry. 2023 May 19;62(11):1786–93. doi: 10.1021/acs.biochem.3c00102 (PMC10249356; doi:10.1021/acs.biochem.3c00102)
Supplement: Supplementary file 1 — bi3c00102_si_001.pdf [file bi3c00102_si_001.pdf]

# Supporting Information for Enhancing substrate specificity of *Clostridium* succinyl-CoA reductase for synthetic biology and biocatalysis

Pascal Pfister*[1], Christoph Diehl*[1], Eric Hammarlund[1], Martina Carrillo[1], Tobias J. Erb[1,2]★

[1] Department of Biochemistry & Synthetic Metabolism, Max Planck Institute for Terrestrial Microbiology, Karl-von-Frisch Str. 10, 35043 Marburg, Germany

[2] SYNMIKRO Center for Synthetic Microbiology, Karl-von-Frisch Str., 14, 35032 Marburg, Germany

*These two authors contributed equally to this work
★corresponding author: toerb@mpi-marburg.mpg.de

## Supplementary Figures

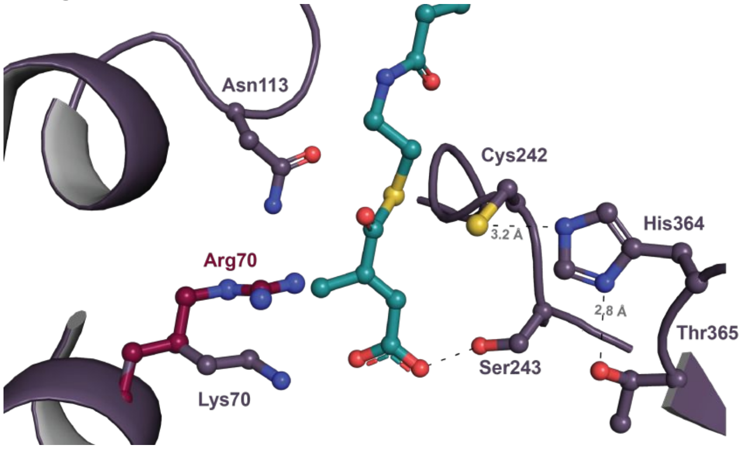


*Figure S1 CkSucD active site with K70R mutation. Peptide residues are presented in violet, mesaconylated intermediates are presented in teal. K70R is colored in red.*

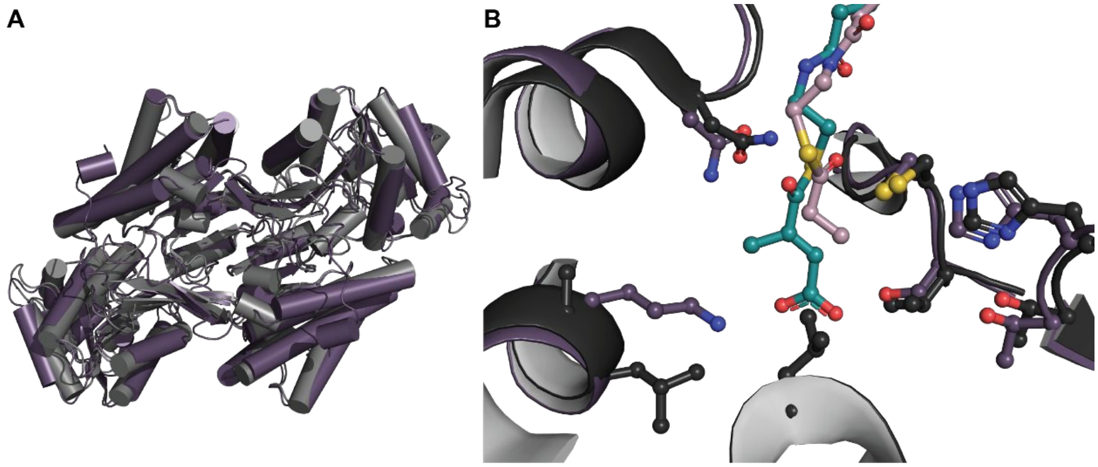


*Figure S2 Comparison of PduP and CkSucD. Secondary structure comparison of CkSucD (violet) and PduP (gray). RMSD value is 1.05 over 302 residues (A). Active site of PduP has different spatial elements. Mesaconyl-C1-CoA (teal) as coordinated in CkSucD would be occluded in PduP by Leu483 (B).*

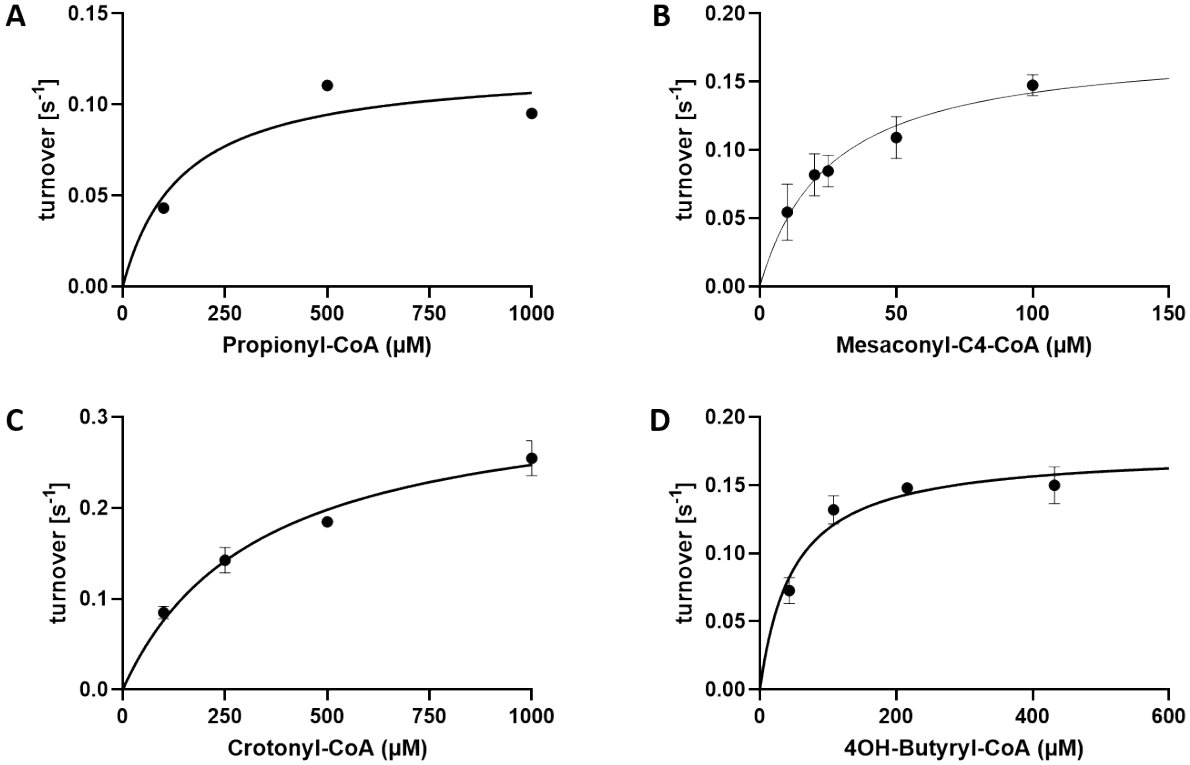


*Figure S3 Michaelis Menten kinetics of CkSucD for different acyl-CoAs: Propionyl-CoA (A), Mesaconyl-C4-CoA (B), Crotonyl-CoA (C), 4OH-Butyryl-CoA.*

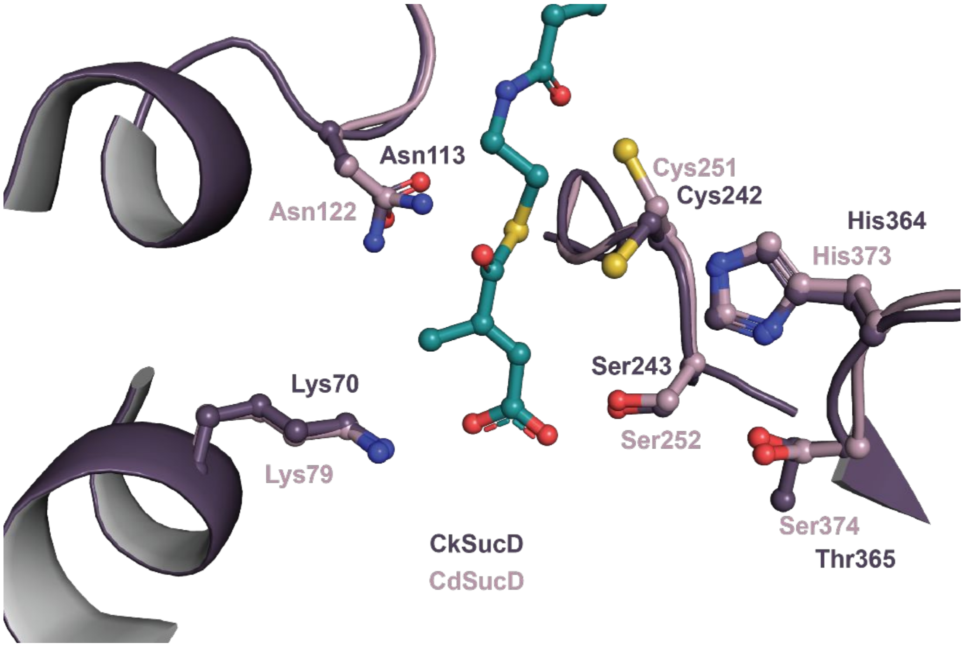


*Figure S4 Overlay of CkSucD and CdSucD active site. CdSucD is presented in violet, CdSucD is presented in rose. mesaconylated intermediates are presented in teal (RMSD of 0.296).*
